# P-53. Trends in Co-administration of Adult Vaccinations in the US Retail Pharmacy Setting

**DOI:** 10.1093/ofid/ofae631.260

**Published:** 2025-01-29

**Authors:** Reiko Sato, Alon Yehoshua, Jeffrey T Vietri, Tianyan Hu, Taryn Pond, Maria J Tort, Jingyan Yang, Verna Welch, Constantina Boikos, Sen Deng, Hao Zheng, Anchita Goswami, Neha Agrawal, Manvi Sharma

**Affiliations:** Pfizer, Inc., Collegeville, Pennsylvania; Pfizer Inc., New York, New York; Pfizer, Inc., Collegeville, Pennsylvania; Pfizer Inc., New York, New York; Pfizer Inc., New York, New York; Pfizer Inc, Collegeville, Pennsylvania; Pfizer and Columbia University Institute for Social and Economic Research and Policy, New York, New York; Pfizer, Collegeville, Pennsylvania; Pfizer Inc., New York, New York; MedAdvisor Solutions, Woburn, Massachusetts; MedAdvisor Solutions, Woburn, Massachusetts; Complete HEOR Solutions (CHEORS), Chalfont, PA, USA, Chalfont, Pennsylvania; Complete HEOR Solutions (CHEORS), Chalfont, PA, USA, Chalfont, Pennsylvania; Complete HEOR Solutions (CHEORS), Chalfont, PA, USA, Chalfont, Pennsylvania

## Abstract

**Background:**

Co-administering multiple vaccines during a single visit can be an efficient strategy to increase vaccine uptake and reduce missed vaccination opportunities. However, the extent to which this is a practice among US adults is not well documented. This study analyzed monthly trends in the co-administration of the US Advisory Committee on Immunization Practices (ACIP) recommended vaccines in the retail pharmacy setting and evaluated the most common combinations of co-administered vaccines.

**Figure 1:**
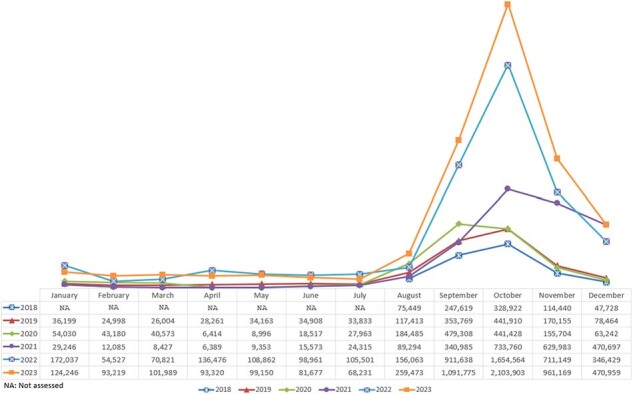
Number of vaccine co-administration by month (August 2018 - December 2023)

**Methods:**

This was a retrospective study using the Adheris Pharmacy Dataset, which includes the majority of retail prescription volume. Adults aged ≥19 years with a record of receiving at least one ACIP-recommended vaccine at a retail pharmacy from August 2018 to December 2023 were included. Vaccine co-administration was defined as receiving two or more vaccines on the same day. Numbers and proportions of vaccine co-administrations were reported by month of administration and age group (19-49, 50-64, ≥65 years). Frequently co-administered vaccines were identified between August 2022 and December 2023. All descriptive analyses were conducted at the visit level.

**Figure 2:**
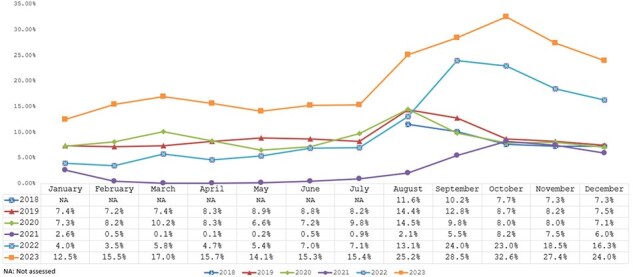
Proportion of vaccine co-administration by month (August 2018 - December 2023)

**Results:**

Overall, there were 16.16 million visits with co-administration of vaccines during the study period, with a range of 0.81 million to 5.55 million annually (**Figure 1**). The proportions of monthly co-administrations showed an upward trend from 2018 (7.3%) to 2023 (32.6%), except for a marked decline in 2021 (**Figure 2**). Co-administration was observed year-round but was considerably higher between September and November across the years and age groups. The number of co-administrations increased with age (**Figure 3**). Among all co-administrations, influenza and COVID-19 vaccines combination was the most common across all age groups (50.0%-78.7%).

**Figure 3:**
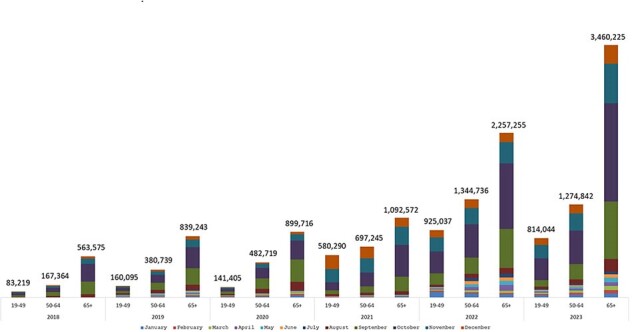
Number of vaccine co-administration by age groups and month (August 2018 - December 2023)

**Conclusion:**

There was an increase in the number and proportion of adult vaccines co-administered in 2022 and 2023 compared to previous years in all age groups. Co-administration of vaccines in the retail pharmacy setting has the potential to improve adult vaccination coverage.

**Disclosures:**

**Reiko Sato, PhD**, Pfizer Inc: employee|Pfizer Inc: Stocks/Bonds (Private Company) **Alon Yehoshua, PharmD, MS**, Pfizer Inc: Employer|Pfizer Inc: Stocks/Bonds (Public Company) **Jeffrey T. Vietri, PhD**, Pfizer Inc.: Employment|Pfizer Inc.: Stocks/Bonds (Public Company) **Tianyan Hu, PhD**, Pfizer: Salary|Pfizer: Stocks/Bonds (Public Company) **Taryn Pond, PharmD**, Pfizer: Employee|Pfizer: Stocks/Bonds (Public Company) **Maria J. Tort, PhD**, Pfizer Inc: Stocks/Bonds (Public Company) **Jingyan Yang, MHS, DrPH**, Pfizer Inc: Employment|Pfizer Inc: Stocks/Bonds (Public Company) **Verna Welch, PhD, MPH**, Pfizer Inc.: Stocks/Bonds (Public Company) **Constantina Boikos, MScPH, PhD**, Pfizer: Full-time employee|Pfizer: Stocks/Bonds (Public Company) **Anchita Goswami, MSc**, Pfizer Inc.: Contracted research **Neha Agrawal, MA**, Pfizer Inc.: Contracted Research **Manvi Sharma, RPh, MBA, PhD**, Pfizer Inc.: Contracted research

